# Cannabis use is associated with pain severity and interference among cancer survivors

**DOI:** 10.21203/rs.3.rs-3126192/v1

**Published:** 2023-07-07

**Authors:** Shannon M. Nugent, Emile Latour, Jeong Lim, Jackilen Shannon, Benjamin J. Morasco

**Affiliations:** Oregon Health & Science University; Oregon Health & Science University; Oregon Health & Science University; Oregon Health & Science University; Oregon Health & Science University

**Keywords:** Cannabis, cancer survivorship, pain management

## Abstract

**Context:**

Cannabis use for symptom management among individuals with cancer is increasingly common.

**Objectives:**

We sought to describe the 1) prevalence and characteristics of cannabis use, 2) perceived therapeutic benefits of cannabis use, and 3) examine how use of cannabis was associated with self-reported pain, mood, and general health outcomes among a representative sample of patients treated at NCI designated OHSU Knight Cancer Institute.

**Methods:**

We conducted a population-based, cross-sectional survey developed in conjunction with 11 other NCI designated cancer centers and distributed to eligible individuals. The survey inquired about characteristics of cannabis use, perception of therapeutic benefits, pain, mood, and general health. Responses were population weighted. We examined the association of cannabis use with self-reported pain, mood, and general health using logistic regression controlling for relevant sociodemographic and clinical characteristics.

**Results:**

523 individuals were included in our analytic sample. 54% endorsed using cannabis at any time since their cancer diagnosis and 42% endorsed using cannabis during active treatment. The most endorsed reasons for use included: sleep disturbance (54.7%), pain (47.1%), and mood (42.6%). We found moderate pain was associated with more than a 2-fold (OR = 2.4 [95% CI = 1.3–4.6], *p* = 0.002) greater likelihood of self-reported cannabis use. Depressed mood or general health were not associated with cannabis use.

**Conclusions:**

In a state with medical and recreational cannabis legislation, a high number of cancer survivors report cannabis use. Those with pain were more likely to use cannabis. Oncologists should be aware of this trend and assess use of cannabis when managing long-term symptoms of cancer and its treatments.

## Introduction

Cancer and its treatments are qualifying conditions in the majority of the 37 states that have legalized medical cannabis.(1) Cannabis use in patients with cancer is common: almost a quarter of individuals with cancer report current cannabis use(2, 3) and more than 90% of cancer survivors view cannabis as potentially beneficial for symptom management and support its legalization.(4)

More than one-third of patients will report moderate to severe pain due to cancer, its treatments, or both.(5, 6) Among patients with cancer who use cannabis, data suggest that approximately 75% use it for symptom management, most commonly pain, nausea, and sleep disruption.(7, 8) Unfortunately, there are a paucity of data about the characteristics of cannabis use and limited evidence about its safety and efficacy for non-cancer pain management (9) with a recent systematic review on cancer pain concluding limited effectiveness of cannabis to improve pain.(10)

Several cross-sectional surveys have linked cannabis use among cancer survivors to the presence of pain.(11, 12) In addition, prior work among cancer survivors, has associated characteristics such as male sex, lack of insurance, younger age and lower education to cannabis use.(3, 11) One study found that those with cancer who use cannabis are more likely to have chronic pain that impacts their daily activities (high impact chronic pain). (12) In addition, those with high impact chronic pain are more likely to perceive a therapeutic benefit of cannabis and less likely to report adverse events related to cannabis compared to those without high impact chronic pain. (12) Data are still lacking about how cannabis use is associated with mood and perception of general health across different time points of cancer survivorship.

Within the current climate of increasing cannabis availability and acceptance, we sought to obtain a more complete and nuanced understanding of cannabis use including frequency of use, modes of use, and reasons for use among a large representative sample of cancer patients. As oncologists increasingly encounter patients using cannabis, knowledge about patient’s use and perceived therapeutic benefit is clinically informative. Thus, our goal was to describe the 1) prevalence and characteristics of cannabis use, 2) perceived therapeutic benefits of cannabis use and 3) examine how use of cannabis was associated with self-reported pain, mood, and general health outcomes among a representative sample of cancer patients treated in Oregon.

## Methods

We conducted a cross-sectional population-based survey. This study was approved by our institutional IRB (OHSU 21714/VA #4679). This project was one of 12 that were funded as part of a National Cancer Institute (NCI) Initiative across NCI designated Cancer Centers.(13)

### Study setting and sample population

The Oregon Health and Science University (OHSU) Knight Cancer Institute (Knight) is the only NCI designated comprehensive cancer center in Oregon. Along with the main campus location, the Knight also has 5 community cancer clinics, and its catchment area is defined as the entire state of Oregon. We obtained a representative sample of patients treated at OHSU Knight from the OHSU Cancer Registry, which collects and maintains information on the demographics, diagnosis, treatment and outcomes of all patients diagnosed with cancer from all of its affiliate clinics.(14) For this study, we included adults (age 21+) who had been diagnosed with any type of cancer, received some treatment at OHSU or its affiliate clinics, and completed treatment within the last 18 months. Data collection occurred between August 2021 and April 2022. There were no study exclusionary criteria.

### Recruitment processes

We used a validated survey method (Dillman Tailored Design Method) to inform the survey procedures.(15) The survey invitations and reminders were sent through electronic health record (EHR)-based electronic messaging. We also added a mailed invitation option for those who were not enrolled in the EHR-based electronic messaging. Patients identified as eligible received electronic or mailed invitations containing (1) a study description, (2) study team contact information, and (3) an opt-out response form. To those who did not opt-out, a letter with a link to the REDCap questionnaire and monetary incentive of $5 gift card was mailed two weeks after the first mailing. Upon request, participants could also receive a paper copy of the questionnaire with a prepaid return envelope. All electronic surveys were completed anonymously. Paper surveys were removed from their envelope and assigned a random code to de-identify.

### Sampling Framework

We used random sampling stratified by rural vs. urban locality defined by patient’s living location using Rural Urban Community Codes (RUCC).(16) Patients were sampled in a ratio of 48.0% urban to 52.0% rural in order to ensure rural representation; actual percentages of eligible patients were 65.0% urban and 35.0% rural. Out of 7,356 potentially eligible patients, a total of 3,725 were sampled.

### Survey development and measures

Through discussion and consensus, we collaboratively developed a set of core data elements along with a national consortium of investigators from 12 funded NCI Comprehensive Cancer Centers in collaboration with ICF Next^™^ (https://www.icf.com), a global marketing and research consultation group. A full copy of the survey instruments can be found at https://epi.grants.cancer.gov/clinical/#initiatives.(13)

#### Current and past use of cannabis.

We inquired about cannabis use in participants’ lifetime, use during cancer treatment, and use in the last 30 days. Among participants who endorsed any use since their cancer diagnosis, follow-up questions assessed cannabis use characteristics including: frequency of cannabis use (e.g. multiple times per day to monthly) and mode of use (e.g. route of cannabis administration such as smoked, tincture, edibles, vaping products, etc.).

#### Therapeutic reasons for use.

We inquired about which symptoms cannabis was used to ameliorate (e.g., pain, chemotherapy induced neuropathy, nausea, lack of appetite, disrupted sleep, to treat-cancer, and mood).

#### Pain and mood.

In addition, while not part of the multi-site data collection, our site included the Pain, Enjoyment, General Activity (PEG) scale which is a 3-item questionnaire assessing pain intensity, interference with enjoyment of life, and interference with activity.(17) The Patient Health Questtionaire-2 (PHQ-2) is a validated 2-item measure of depression severity.(18) Higher scores on the PEG and PHQ-2 indicate more severe symptoms.

#### General Health.

We included a single-question inquiring about perception of general health in which the participant was asked to rate their health from Excellent to Poor on a 5 point Likert scale.(19)

#### Opioid Questions.

We also assessed whether individuals were using prescription opioids (current, in the last 3 months, more than three months in the past, or never). Response to these questions was included as a dichotomous covariate (current versus all other time points) to represent current opioid use in the regression models.

#### Sociodemographic.

Information about age, sex, gender identity, race/ethnicity, income, education level, and insurance status were obtained via self-report. We did not obtain data on cancer type and treatment.

### Data Analysis

Population survey weights were used to ensure that the sample of survey respondents were representative of the Oregon population and to reduce potential survey bias. Rural/urban status was not collected from survey respondents, and so equal probability of selection was assigned to each respondent. From nonresponse analysis, significant differences in the response rates by age category and race were detected. A raking algorithm was used to iteratively adjust the base weights to population totals using age, sex, race, and ethnicity to account for potential nonresponse bias. Appropriate statistical adjustment was used to incorporate the estimated weights into all analyses.

Descriptive statistics were used to summarize the survey responses (means and standard deviations for continuous variables; counts and percentages for categorical). Participants were divided into two groups based on whether they endorsed cannabis use after their cancer diagnosis. T-tests and chi-square tests were used to compare the demographic characteristics based on self-reported cannabis use status. Descriptive statistics were also used to summarize barriers to using cannabis, therapeutic reasons for using, characteristics of use (e.g., frequency, amount, routes of administration), and adverse events experienced.

We used multivariable logistic regression modelling to examine the relationships between cannabis use (as the outcome) and pain, general health, and mood. Each of these main effects of interest were modelled separately to independently assess their association with cannabis use. These models also included additional covariates to adjust for demographic and personal characteristics: age, race/ethnicity, sex, education level completed, household income, employment status, prior cannabis use, opioid use, and health coverage. Models were assessed for multicollinearity, and no issues were found. Statistical analyses used R: A Language and Environment for Statistical Computing.(20) We used the srvyr(21) and survey packages(22) for weighted analysis of the population survey data. We did not use imputation in the weighted analysis. A missing category is shown in the tables. For the regression models, those missing outcome (cannabis use) were excluded, and a missing category was used for the predictors.

## Results

**Figure 1:**
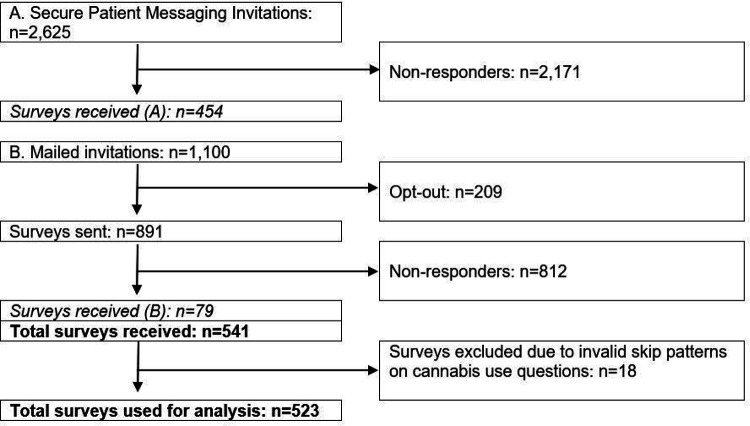
Participant Flow Chart

We reached out to 3,725 unique individuals of 7,356 potentially eligible patients (2,625 via EHR messaging and 1,100 via mailed invitation; Fig. 1). A total of 541 patients met study inclusion criteria and answered the survey (14% response rate); due to missing data or skip pattern failures, 523 were retained for the final analytic sample. We examined sociodemographic variables (age, sex at birth, race and ethnicity) that were associated with non-response (data not shown). Individuals who were more likely to complete the survey were non-white and older than 80 years.

The population weighted and unweighted characteristics of our enrolled sample are summarized in Table 1. Fifty-four percent of our participants endorsed using cannabis at some point after their cancer diagnosis (hereafter referred to as cannabis users). Forty-two percent endorsed using during treatment and an additional 26.6% reported considering cannabis use at some point since their cancer diagnosis. In addition, 71.8% endorsed using cannabis at any time over the course of their life. In unadjusted analyses, the following sociodemographic characteristics were associated with cannabis use at any time following diagnosis: younger age (61.4 vs 65.8, *p* = 0.001), driven by a higher percentage of 18- to 44-year-olds who reported cannabis use (16.3% vs. 8.1%). A significantly higher proportion of those who used cannabis endorsed “finding it difficult to get by on my present income” (12.4% vs 4.4%, *p* = 0.016). Finally, there was an association with primary source of health insurance and cannabis use with a notable difference in the percentage of those that used cannabis and had Medicaid compared to those that did not use cannabis and had Medicaid (15.1% vs 5.7%, p = 0.005). There was also a higher percentage with private health insurance plans among those who did not use cannabis compared to those who did use cannabis (38.8% vs 31.8%, p = 0.005). There were no other significant differences based on demographic characteristics between the two groups (Table 1).

Differences between the two groups on measures of pain, perception of general health and mood, are displayed in Table 2. Of these variables, higher pain severity, pain interference, and depressive symptomatology were all associated with cannabis use in univariate analyses (all *p* ≤ 0.001). General health was not associated with endorsement of cannabis use.

**Figure 2:**
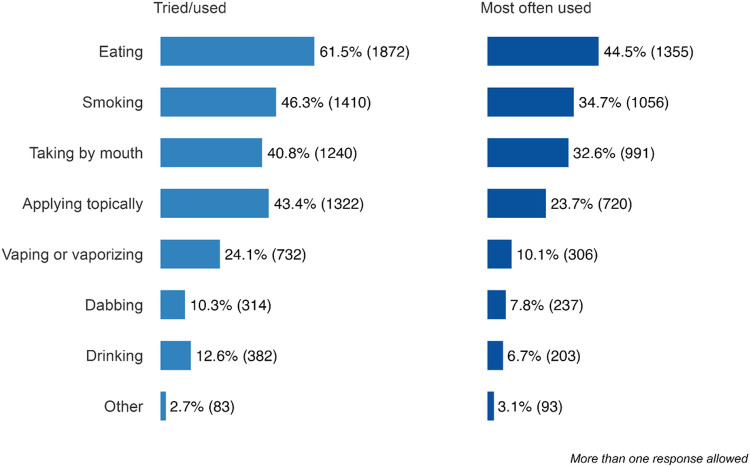
Self-reported routes of cannabis administration

The most commonly endorsed reasons for current cannabis use included: sleep disturbance (54.7%, n = 1663), pain (47.1%, n = 1432), mood changes, stress, anxiety or depression (42.6%, n = 1296), and recreation (35.6%, n = 1083; Table 3). Among those who reported using cannabis during or after cancer treatment, the majority did so multiple times per week or more (Table 4). The four most preferred routes of administration endorsed were: eating (i.e., candy, brownies) (44.5%, n = 1355), smoking (34.7%, n = 1056), taking by mouth (e.g., tincture, pills) (32.6%, n = 991), and applying topically (23.7%, n = 720; Fig. 2).

**Figure 3:**
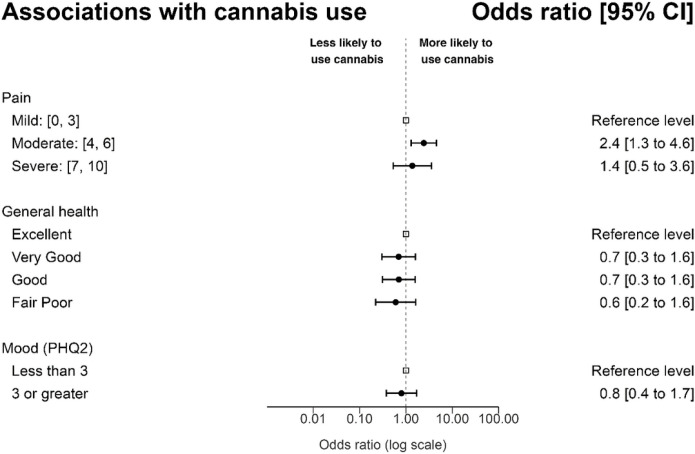
Forest Plot of Regression Analyses

Logistic regression analyses were conducted to examine variables significantly associated with cannabis use at anytime since cancer diagnosis. Covariates included in these analyses were age, sex, race/ethnicity, education level completed, household income, occupational status, prior cannabis use, opioid use, and health coverage status. Results indicate that participants with moderate past week average pain severity were more likely to use cannabis (OR = 2.4 [95% CI = 1.3–4.6], *p* = 0.002). In this model, non-Hispanic ethnicity and prior cannabis use were also associated with an increased likelihood of cannabis use (p < 0.001). A second logistic regression was conducted to examine the extent to which self-rated health status was associated with current cannabis use; this model controlled for the same variables. Results did not identify a relationship between self-reported poor or fair health (versus excellent health) and cannabis use (OR = 0.6 [95% CI = 0.2–1.6], *p* = 0.316). A final model examined the extent to which depressive symptomatology was associated with cannabis use. After controlling for covariates, depressed mood (as indicated by a score of 3 or greater on the PHQ-2) was not associated with an increased risk of cannabis use (OR = 0.8 [95% CI = 0.4–1.7], *p* = 0.566, Fig. 3).

## Discussion

Cancer survivorship is often fraught with management of ongoing symptoms, including pain, depression, and impairments in general health. With changes in legalization, cancer survivors may be increasingly using cannabis as a strategy for managing symptoms, but the extent to which cannabis is effective is unclear. A more complete picture of therapeutic reasons for use, routes of administration and patterns of use are clinically important and can help oncologic clinicians evaluate potential risks and benefit. In a representative sample of individuals receiving care at an NCI designated cancer center in Oregon, we found that 54% of patients used cannabis at some point after their diagnosis and 42% used during treatment. This prevalence is higher than the national prevalence of cannabis use among cancer survivors in 2020, which was estimated to be about 8% and the 12.4% that was reported in by a survey conducted by an NCI cancer center spanning multiple states (Minnesota, Florida, and Arizona).(11) A likely driver of the notably higher prevalence in our sample is the legal status in Oregon, which has allowed medical cannabis since 1998 and recreational cannabis (use for anyone 21+) since 2015.(23)

Nuanced information about how patients are using cannabis is important to inform clinical conversations around patient safety and to screen for potential risks. In our sample, about a quarter of individuals reported using cannabis daily and an additional 14% used multiple times per day. Daily or more than daily use has been linked to the development of cannabis use disorder, albeit in a younger population.(24) In addition, we found that consuming cannabis via edibles was the most regularly used route of administration (endorsed by about 44% of participants) followed by smoking (35%). These frequencies and preferred roues of cannabis use are similar to what has been documented in other research.(3) Inhalation of cannabis has been linked to bronchial infections, which may have particular relevance to those with lung or upper aerodigestive tract cancers.(24, 25) These findings point to particular areas of additional clinical inquiry needed to effectively counsel patients using cannabis on potential harm.

Finally, we found that those with moderate pain severity were 2.4 times more likely than those without pain to endorse using cannabis sometime after their cancer diagnosis, when controlling for covariates. This finding is somewhat consistent with other recently published data from another NCI Designated Cancer center, that cannabis users were more likely to have high impact chronic pain (12) (defined as pain that limits life or work activities on most day or every day in the last three months). Yet, interestingly in our data, those with the most severe pain were not more likely than those with no pain to use cannabis. In addition, depressive symptoms and general health were not found to be associated with cannabis use in adjusted analyses. In the context of long-term cancer symptom management, further inquiry could be focused on understanding the prospective effect of cannabis on self-reported symptoms such as pain, mood and general health. Such information is essential for oncology care providers to counsel patients effectively on cannabis use (26)

There are several limitations which should be considered when interpreting the results from this study. First, these data are cross sectional so temporal relationships between variables cannot be inferred. Second, we did not collect data on cancer type and treatment, which limits our ability to contextualize these findings based on cancer variables. Finally, while we tried to be explicit in our recruitment materials that we were interested in perceptions about cannabis use, we may have obtained a response bias skewing toward those who were using cannabis.

We found about half of cancer survivors used cannabis at some point following their diagnosis, with many choosing edibles and using multiple times per week. In our sample, the presence of moderate pain placed individuals at a higher likelihood to use cannabis, while the presence of depressed mood or poor health did not. Clinicians should assess for cannabis use status among all patients during and after cancer treatment. Prospective research is needed to understand the clinical benefits and harms from use.

## Figures and Tables

**Table 1:** Participant characteristics by endorsement of cannabis use anytime since their cancer diagnosis

Category	Level	Weighted				Unweighted			
		Overall	Yes	No	p	Overall	Yes	No	p
n	-	7218	3972	3246		523	287	236	
Age, mean (SD)	-	63.38 (16.36)	61.40 (16.68)	65.80 (15.66)	0.001	63.72 (17.34)	61.42 (17.68)	66.51 (16.52)	0.001
Age, categorical, % (n)					0.006				0.002
	18 to 44	12.6 (910)	16.3 (646)	8.1 (264)		14.0 (73)	18.1 (52)	8.9 (21)	
	45 to 54	35.3 (2549)	35.5 (1409)	35.1 (1140)		33.1 (173)	33.4 (96)	32.6 (77)	
	65 to 74	33.5 (2420)	33.7 (1340)	33.3 (1080)		32.7 (171)	32.8 (94)	32.6 (77)	
	75 or older	18.6 (1340)	14.5 (577)	23.5 (762)		20.3 (106)	15.7 (45)	25.8 (61)	
Sex assigned at birth, % (n)					0.707				0.722
	Male	44.2 (3187)	45.5 (1807)	42.5 (1380)		41.5 (217)	42.9 (123)	39.8 (94)	
	Female	52.7 (3804)	52.7 (2092)	52.7 (1712)		54.5 (285)	54.7 (157)	54.2 (128)	
	(Missing)	3.1 (226)	1.8 (73)	4.7 (154)		4.0 (21)	2.4 (7)	5.9 (14)	
Gender, % (n)					0.437				0.444
	Male	43.4 (3129)	44.8 (1779)	41.6 (1350)		40.7 (213)	42.2 (121)	39.0 (92)	
	Female	52.3 (3777)	51.6 (2050)	53.2 (1727)		54.1 (283)	53.7 (154)	54.7 (129)	
	Transgender	0.2 (11)	0.3 (11)	0.0 (0)		0.2 (1)	0.3 (1)	0.0 (0)	
	None of the above	0.4 (28)	0.7 (28)	0.0 (0)		0.4 (2)	0.7 (2)	0.0 (0)	
	(Missing)	3.8 (273)	2.6 (104)	5.2 (169)		4.6 (24)	3.1 (9)	6.4 (15)	
Race x Ethnicity, % (n)					0.191				0.168
	Hispanic or Latino	2.7 (194)	3.2 (129)	2.0 (65)		2.3 (12)	2.8 (8)	1.7 (4)	
	Not Hispanic or Latino, White	89.5 (6458)	90.5 (3594)	88.3 (2864)		88.5 (463)	89.9 (258)	86.9 (205)	
	Not Hispanic or Latino, Black	0.3 (25)	0.0 (0)	0.8 (25)		0.4 (2)	0.0 (0)	0.8 (2)	
	Not Hispanic or Latino, Asian	0.4 (26)	0.0 (0)	0.8 (26)		0.4 (2)	0.0 (0)	0.8 (2)	
	Not Hispanic or Latino, Other	3.5 (251)	2.9 (114)	4.2 (137)		3.8 (20)	3.1 (9)	4.7 (11)	
	(Missing)	3.7 (264)	3.4 (136)	3.9 (128)		4.6 (24)	4.2 (12)	5.1 (12)	
Income, % (n)					0.081				0.082
	Less than $35,000	21.8 (1570)	25.1 (996)	17.7 (575)		21.8 (114)	25.1 (72)	17.8 (42)	
	$35,000 to $74,999	25.6 (1848)	27.0 (1073)	23.9 (775)		25.4 (133)	26.8 (77)	23.7 (56)	
	$75,000 or more	42.1 (3041)	38.9 (1 546)	46.0 (1494)		41.5 (217)	38.3 (110)	45.3 (107)	
	(Missing)	10.5 (759)	9.0 (357)	12.4 (401)		11.3 (59)	9.8 (28)	13.1 (31)	
Feelings about household income, % (n)					0.016				0.022
	Living comfortably on present income	49.9 (3600)	47.3 (1877)	53.1 (1722)		49.3 (258)	46.7 (134)	52.5 (124)	
	Getting by on present income	27.3 (1971)	28.1 (1114)	26.4 (856)		27.3 (143)	28.2 (81)	26.3 (62)	
	Finding it difficult on present income	8.8 (636)	12.4 (492)	4.4 (144)		8.8 (46)	12.2 (35)	4.7 (11)	
	Finding it very difficult on present income	5.7 (409)	6.4 (254)	4.8 (155)		5.5 (29)	6.3 (18)	4.7 (11)	
	(Missing)	8.3 (602)	5.9 (235)	11.3 (368)		9.0 (47)	6.6 (19)	11.9 (28)	
Occupation Status, % (n)					0.087				0.083
	Employed	31.0 (2236)	29.1 (1155)	33.3 (1081)		30.8 (161)	29.3 (84)	32.6 (77)	
	Unemployed	3.0 (219)	3.8 (149)	2.2 (70)		3.3 (17)	4.2 (12)	2.1 (5)	
	Homemaker	2.9 (208)	3.5 (139)	2.1 (69)		2.9 (15)	3.5 (10)	2.1 (5)	
	Student	1.1 (82)	1.4 (57)	0.8 (25)		1.3 (7)	1.7 (5)	0.8 (2)	
	Retired	44.6 (3221)	42.1 (1671)	47.8 (1550)		44.0 (230)	41.1 (118)	47.5 (112)	
	Disabled	11.4 (821)	14.6 (579)	7.5 (242)		10.9 (57)	13.9 (40)	7.2 (17)	
	Other (Specify):	1.9 (138)	2.5 (98)	1.2 (39)		1.9 (10)	2.4 (7)	1.3 (3)	
	(Missing)	4.1 (294)	3.1 (123)	5.3 (170)		5.0 (26)	3.8 (11)	6.4 (15)	
Highest level of education completed, % (n)					0.273				0.297
	Less than high school	2.0 (146)	2.1 (83)	1.9 (63)		1.9 (10)	2.1 (6)	1.7 (4)	
	High school diploma	8.9 (645)	9.3 (371)	8.5 (274)		9.0 (47)	9.4 (27)	8.5 (20)	
	Some college	25.8 (1865)	29.4 (1166)	21.5 (698)		25.2 (132)	28.6 (82)	21.2 (50)	
	Vocational training or 2-year degree	3.9 (283)	4.6 (184)	3.1 (99)		3.8 (20)	4.5 (13)	3.0 (7)	
	4-year college degree or more	56.0 (4039)	52.5 (2084)	60.2 (1955)		55.8 (292)	52.6 (151)	59.7 (141)	
	(Missing)	3.3 (239)	2.1 (84)	4.8 (155)		4.2 (22)	2.8 (8)	5.9 (14)	
Has health coverage, % (n)					0.532				0.433
	Yes	93.3 (6735)	93.6 (3719)	92.9 (3015)		92.4 (483)	92.7 (266)	91.9 (217)	
	No	3.2 (228)	3.6 (144)	2.6 (84)		3.3 (17)	3.8 (11)	2.5 (6)	
	(Missing)	3.5 (255)	2.7 (109)	4.5 (146)		4.4 (23)	3.5 (10)	5.5 (13)	
Primary source of health coverage, % (n)		0.005			0.005				0.004
	Private plan through employer, family member, or buys for self	34.9 (2522)	31.8 (1261)	38.8 (1260)		34.4 (180)	31.7 (91)	37.7 (89)	
	Medicare	40.8 (2943)	39.2 (1 558)	42.7 (1385)		40.7 (213)	38.7 (111)	43.2 (102)	
	Medicaid or other state program	10.9 (785)	15.1 (599)	5.7 (185)		10.7 (56)	15.0 (43)	5.5 (13)	
	TRICARE (formerly CHAMPUS), VA, or Military	3.9 (282)	4.6 (183)	3.1 (100)		3.6 (19)	4.2 (12)	3.0 (7)	
	Some other source	1.1 (80)	1.7 (66)	0.4 (13)		11 (6)	1.7 (5)	0.4 (1)	
	(Valid skip)	6.7 (483)	6.4 (253)	7.1 (230)		7.6 (40)	7.3 (21)	8.1 (19)	
	(Missing)	1.7 (123)	1.3 (52)	2.2 (72)		17 (9)	14 (4)	2.1 (5)	

Differences between the two groups on measures of pain, perception of general health and mood, are displayed in Table 2. Of these variables, higher pain severity, pain interference, and depressive symptomatology were all associated with cannabis use in univariate analyses (all *p* ≤ 0.001). General health was not associated with endorsement of cannabis use.

**Table 2:** Pain, General Health and Depression by Cannabis Use Since Diagnosis

Category	Level	Weighted				Unweighted			
		Overall	Yes	No	p	Overall	Yes	No	p
n		7218	3972	3246		523	287	236	
Pain on average in the past week, % (n)					< 0.001				0.001
	Mild: [0, 3]	64.4 (4645)	58.9 (2339)	71.1 (2306)		63.9 (334)	58.9 (169)	69.9 (165)	
	Moderate: [4, 6]	22.5 (1623)	28.7 (1140)	14.9 (484)		22.2 (116)	28.2 (81)	14.8 (35)	
	Severe: [7, 10]	8.0 (576)	9.8 (390)	5.8 (187)		8.2 (43)	10.1 (29)	5.9 (14)	
	(Missing)	5.2 (373)	2.6 (104)	8.3 (269)		5.7 (30)	2.8 (8)	9.3 (22)	
During the past week, how has pain interfered with enjoyment of life, % (n)					< 0.001				< 0.001
	Mild: [0, 3]	66.0 (4764)	60.2 (2389)	73.2 (2374)		65.6 (343)	60.3 (173)	72.0 (170)	
	Moderate: [4, 6]	19.0 (1370)	24.7 (982)	11.9 (388)		18.7 (98)	24.4 (70)	11.9 (28)	
	Severe: [7, 10]	9.5 (684)	11.8 (467)	6.7 (216)		9.6 (50)	11.8 (34)	6.8 (16)	
	(Missing)	5.6 (401)	3.4 (134)	8.2 (267)		6.1 (32)	3.5 (10)	9.3 (22)	
During the past week, how has pain interfered with general activity, % (n)					0.001				0.001
	Mild: [0, 3]	66.3 (4786)	61.8 (2454)	71.8 (2332)		66.0 (345)	61.7 (177)	71.2 (168)	
	Moderate: [4, 6]	17.5 (1260)	21.0 (834)	13.1 (426)		17.0 (89)	20.6 (59)	12.7 (30)	
	Severe: [7, 10]	12.1 (872)	16.2 (643)	7.0 (228)		12.2 (64)	16.4 (47)	7.2 (17)	
	(Missing)	4.2 (300)	1.0 (41)	8.0 (259)		4.8 (25)	1.4 (4)	8.9 (21)	
General health, % (n)					0.118				0.113
	Excellent	8.5 (613)	9.9 (394)	6.7 (219)		8.6 (45)	10.1 (29)	6.8 (16)	
	Very Good	28.1 (2026)	24.7 (980)	32.3 (1047)		28.1 (147)	24.7 (71)	32.2 (76)	
	Good	47.1 (3400)	49.7 (1974)	43.9 (1425)		46.5 (243)	49.5 (142)	42.8 (101)	
	Fair Poor	12.9 (928)	14.4 (570)	11.0 (357)		12.6 (66)	13.9 (40)	11.0 (26)	
	(Missing)	3.5 (251)	1.4 (54)	6.1 (197)		4.2 (22)	1.7 (5)	7.2 (17)	
PHQ-2 Score^a^ % (n)					0.001				0.001
	Less than 3	83.5 (6030)	86.2 (3425)	80.3 (2605)		82.8 (433)	85.7 (246)	79.2 (187)	
	3 or greater	9.8 (704)	10.7 (424)	8.6 (280)		9.8 (51)	10.8 (31)	8.5 (20)	
	(Missing)	6.7 (483)	3.1 (123)	11.1 (360)		7.5 (39)	3.5 (10)	12.3 (29)	

a Note: PHQ-2 scores of 3 or greater are indicative of the likely presence of a Major Depressive Disorder (and would warrant further clinical assessment)

**Table 3:** Self-Reported Reasons for Cannabis Use

Reasons	%^a^ (population weighted n)
Difficulty sleeping	54.7 (1663)
Pain	47.1 (1432)
Mood changes, stress, anxiety, or depression	42.6 (1296)
Used recreationally or for enjoyment	35.6 (1083)
Lack of appetite	24.5 (747)
Digestive problems (e.g., nausea, vomiting, diarrhea, constipation)	23.1 (702)
Used as treatment for cancer	16.6 (506)
Neuropathy (numbness or tingling)	16.2 (491)
Lack of energy	7.8 (237)
Sweating symptoms (e.g., hot flashes, night sweats)	6.2 (188)
Difficulty concentrating	5.0 (1 52)
Lack of sexual interest of activity	3.5 (107)
Skin problems	0.9 (28)

a Note: participants were informed to "select all that apply" so numbers exceed 100%.

**Table 4:** Frequency of cannabis use during and after treatment

During treatment, how often cannabis used (n = 3042)	% (population weighted n)
Once a month or less	17.0 (518)
A few times a month	10.5 (318)
A few times a week	22.9 (696)
Daily or almost daily	29.5 (898)
More than once a day	19.3 (587)
**After treatment, how often cannabis used (n = 2339)**	
More than once a day	13.6 (413)
Daily or almost daily	24.3 (740)
A few times a week	21.3 (647)
A few times a month	13.2 (402)
Once a month or less	7.7 (236)
Only tried it once or twice	10.2 (310)

## Data Availability

Data can be shared upon request.

## Key Message

This study describes results from a population-based, cross-sectional survey examining cannabis use among individuals treated at an NCI designated cancer center. We found that cannabis use was prevalent following cancer treatment (54%). Cannabis use was associated with the presence of moderate pain (when controlling for relevant clinical and sociodemographic variables).
